# Supporting Artificial Social Intelligence With Theory of Mind

**DOI:** 10.3389/frai.2022.750763

**Published:** 2022-02-28

**Authors:** Jessica Williams, Stephen M. Fiore, Florian Jentsch

**Affiliations:** ^1^Team Performance Laboratory, University of Central Florida, Institute for Simulation and Training, Orlando, FL, United States; ^2^Cognitive Sciences Laboratory, University of Central Florida, Institute for Simulation and Training, Orlando, FL, United States

**Keywords:** theory of mind, artificial social intelligence, human-agent interaction, social signal processing, social intelligence and cognition, transparency

## Abstract

In this paper, we discuss the development of artificial theory of mind as foundational to an agent's ability to collaborate with human team members. Agents imbued with artificial social intelligence will require various capabilities to gather the social data needed to inform an artificial theory of mind of their human counterparts. We draw from social signals theorizing and discuss a framework to guide consideration of core features of artificial social intelligence. We discuss how human social intelligence, and the development of theory of mind, can contribute to the development of artificial social intelligence by forming a foundation on which to help agents model, interpret and predict the behaviors and mental states of humans to support human-agent interaction. Artificial social intelligence will need the processing capabilities to perceive, interpret, and generate combinations of social cues to operate within a human-agent team. Artificial Theory of Mind affords a structure by which a socially intelligent agent could be imbued with the ability to model their human counterparts and engage in effective human-agent interaction. Further, modeling Artificial Theory of Mind can be used by an ASI to support transparent communication with humans, improving trust in agents, so that they may better predict future system behavior based on their understanding of and support trust in artificial socially intelligent agents.

## Introduction

Robots, virtual assistants, and other kinds of agents imbued with artificial intelligence (AI) have been, and will continue to be, increasingly implemented across many industries, such as healthcare, military, and transportation, to name a few of the many areas that are being transformed by advances in these technologies (Misuraca et al., 2020). AI has also been useful in numerous different applications, from healthcare, such as evaluating and determining treatment in orthopedic surgeries (Haleem et al., 2020), to business, where machine learning drives marketing decisions (Cuzzolin et al., 2020; Rai, 2020). Though artificial intelligence is already applied to a broad range of domains, most implementations are generally static, deterministic models that function as an expert system (a system of rules defined by a human through programmed “if-then” functions), rather than what would be considered true artificial intelligence that is comparable to human intelligence (Kaplan and Haenlein, 2019).

Recent technological advances have emerged that attempt to use cognitive models to build human-inspired intelligence in artificial agents. These tend to rely on black-box models such as machine learning, neural networks, deep learning (LeCun et al., 2015), or model-based reinforcement learning (Gao et al., 2019). The methods these employ to fit data and identify patterns are opaque, providing little transparency into the internal processes by which they make these determinations (Calder et al., 2018). Thus, the reasons for which they exhibit certain behaviors are often not easily interpretable (Calder et al., 2018; Rai, 2020). Humans are not capable of replicating the processes machine learning models use to discover patterns in data (Misuraca et al., 2020), nor how these models rationalize their determinations about a system (Bennett and Maruyama, 2021). This has led to the development of new areas of research, such as “explainable artificial intelligence” (XAI; Gunning et al., 2019). Here, the goal is to either, ensure underlying decision processes are less opaque (Fernandez et al., 2019), or create techniques to translate, for example, ML outputs, into something understandable (Chatzimparmpas et al., 2020). In this paper we do not focus on XAI, but, rather, discuss the implications of this problem for human-machine teaming and how ASI can help address it.

The lack of transparency in these models contributes to reduced trust along a number of dimensions. Most simply, when users are not able to understand the behaviors or decisions they observe, there is a loss of trust (Miller, 2019). This can decrease acceptance of agent input, thereby reducing agent efficacy in coming to reliable determinations consistently and justifiably (Horst et al., 2019). This is problematic because trust is recognized as a significant and desirable characteristic of human-agent interactions (Jacovi et al., 2021). Understanding agent decisions fosters the development of trust and is fundamental to supporting interaction between users and artificial agents (Papagni and Koeszegi, 2021). Ensuring users possess accurate perceptions of agent capability and intent, and that they are informed of contextually-relevant constraints and other appropriate system reliability-related knowledge, will become increasingly important as the autonomous capabilities of AI systems advance (Lyons, 2013).

If a human does not have trust in an agent, they will not delegate tasks to it regardless of whether the agent is truly capable and will lead to misuse of the AI system. Further, over-reliance on the system can lead to human complacency and failing to detect AI system errors (Alonso and De La Puente, 2018). The miscalibration of human trust in AI, and resulting inappropriately reliance on the agent, will cause decreased performance in human-agent teams (Alonso and De La Puente, 2018). Thus, trust is a key factor in human-agent interaction and influences whether a human will rely upon the system (Lyons, 2013). As noted above, trust in AI systems is closely related to the level of understandability and predictability of a system (Akula et al., 2019). Essentially, the humans understanding of the behaviors and decisions of a system influence the trust a human will place in the system (Akula et al., 2019).

To promote trust, we suggest that agents will need to be imbued with artificial social intelligence (ASI) that can help calibrate outputs of their internal models in human-understandable ways. More generally, effective human-agent collaboration requires the agent possess social intelligence and the communicative and social skills necessary for agents to maintain the effective exchange of social information (Fiore et al., 2013; Wiltshire et al., 2014). The development of models of artificial social intelligence will require interdisciplinary collaboration between computer science, AI, and machine learning researchers, and social science researchers, such as philosophy, cognitive psychology/science, computer science, and social psychology (Miller, 2019).

A well-established component of social intelligence in humans is Theory of Mind (ToM). This is generally seen as the ability to recognize and attribute mental states, beliefs, desires, and intentions to others. As a core process in social cognition, it is fundamental to our ability to interact (Chen et al., 2021b). Said another way, social intelligence can be considered the manifestation of Theory of Mind in adults. A well-developed ToM is strongly correlated with the ability to correctly judge the trustworthiness of others because ToM allows us to perform behavioral predictions about actions that others will take based on our perceptions of them and our knowledge of the situation's context (Vinanzi et al., 2019). Further, ToM enables discovery of false or incomplete beliefs and knowledge, which can be corrected through interventions (Oguntola et al., 2021). Because it is a foundational element of human social intelligence, we argue that ToM is crucial for developing AI systems that effectively interact with humans in teams or in multi-agent systems (Oguntola et al., 2021).

A critical research need is identification of how to create agents capable of theory of mind. One approach could involve adapting models of human ToM to serve as an analog in developing an agent that possesses an Artificial Theory of Mind (AToM). With this, the ASI agent could model a human user's ToM using input humans incorporate, such as verbal and non-verbal social cues. Reciprocally, an ASI agent can draw from their AToM to help their teammate develop a corresponding model of agent theory of mind. As described above, the provision of transparent information can help humans understand and interpret agent behavior. Transparency helps humans to better understand the agent's actions and predict the future behavior of the AI system more accurately (Riedl, 2019). Our point here is that ASI can use its AToM to determine how to best communicate information in a way that maintains agent-to-human transparency (Lyons, 2013), and that is appropriate for a given humans experience level (e.g., expert vs. non-expert users). Through transparent interactions, an agent revises its theory of mind about another and understand how the other came to a decision or why they took an action (Riedl, 2019). Overall, then, transparency in agent decision-making processes, as well as supporting interpretable and understandable agent behaviors, will foster the development of trust in humans. In sum, ASI enabled agents can partake in a dynamic give-and-take with their teammates where they draw from their AToM to both understand their human counterparts but also intervene to improve overall team understanding and function. We next illustrate this with a concrete example.

Consider a situation in which a human-agent team is part of internationally-deployed forces. It is not uncommon for such teams to experience problems from cultural differences in their operational environment. Here, for example, they could be tasked with de-escalating an interaction between one of their team members and a civilian native to the location they are stationed. Socio-cultural norms and values are likely extremely different from the cultures represented in the human-agent team (e.g., the customs related to respect may be significantly different or more explicitly communicated). An ASI could perceive the tenor of the interaction by monitoring the social signals and developing a ToM of the relevant parties. From this, the ASI could assist in the interaction by facilitating communication using its capability to account for each agent's knowledge, beliefs, desires, and intentions. Further, the agent could use its own prior experiences, modeled after episodic memories (Vinanzi et al., 2019), to make more accurate predictions about what might be the best way to remedy the situation. Given that such training is standard for military personnel (e.g., Brown et al., 2019), it might even be feasible to train an ASI for such scenarios. From this, the ASI could maintain richer contextually-based knowledge and teammate descriptors/evaluations/characteristics, allowing it to identify mental model mismatch and predict how those incongruencies may hinder interaction. Overall, then, a socially intelligent agent could use its models of the culture foreign to the forces, the individuals based on their interactions, and historical data of the team members involved, to generate conclusions and recommendations.

The above example provided a relevant illustration of ASI. Just as critical, though, is consistency in ASI behavior. Humans are more likely to trust agents when they understand and can predict the behavior of the system based on prior interactions with the system. Consistency and clarity in explanations build trust in the agent's determinations. Thus, it is important to maintain not only transparency and interpretability in the explanations, but also consistency in agent behavior. This will help reinforce the models of the ASI's AToM in their human counterparts. When an ASI agent is transparent and provides information about itself to human team members, humans can better understand the intentions behind agent actions or the logic behind determinations (Lyons, 2013). Similarly, providing an ASI with transparent information related to human behavior will be essential to informing the agent's AToM. This can be employed to facilitate more accurate machine predictions and responses, and effective human-agent interaction—particularly in complex social situations. An AToM model would start with a basic initial state that is iteratively updated with each interaction, which is integrated into, and further trains, the model through the outcomes from subsequent interactions with a given human. Facilitating these interactions and processes will require receiving, interpreting, and generating social information arising from the interaction (Alonso and De La Puente, 2018).

In sum, we advance interdisciplinary discussions surrounding the development of an Artificial Social Intelligence (ASI) drawing from, and relating, concepts from the social, cognitive, and computer sciences to identify the interdisciplinary contributions and domain-specific requirements necessary for an Artificial Theory of Mind. This article is not a survey of existing approaches to machine learning, explanation in AI, or interpretability in AI, nor will it discuss specific modeling techniques. Such a review is beyond the scope of this work. Nonetheless, when appropriate, we touch upon machine learning and modeling techniques used in prior research that are relevant to our discussion of the development of social intelligence and technologies. In the following pages, we provide a review of social intelligence and theory of mind in humans, and conceptually adapt and apply theories to artificial intelligence. From this, we lay the foundation for computer science and AI researchers to understand what the constituent elements of an AToM are to support building a robust, cognitive model in ASI. Within these sections we also include interdisciplinary considerations for the design and building of an ASI with a particular emphasis on AToM.

## Social Intelligence in Humans

The separability of social elements from other types of intelligence has been widely acknowledged since Thorndike's distinction of social from mechanical and abstract intelligence (Thorndike, 1920)—this work is often pointed to as one of the earliest instances of attempting to define social intelligence. Definitions of social intelligence differ in notable ways—some emphasize cognitive components, others focus on behavior, while others consider psychometric methods to measure social skills (Silvera et al., 2001). In addition to disagreements in the operational definition of social intelligence, attempts to establish reliable methods to quantify social intelligence have also had problems. These are related to validity and reliability, particularly in non-verbal measures of social intelligence, and have demonstrated a potential for self-report biases (Silvera et al., 2001). These complications in definition and measurement arise because social intelligence is multifaceted in nature, involving a number of components that have been identified in prior research (Silvera et al., 2001). Because of this, some suggest that social intelligence should be understood through the abilities and skills needed for successful social interaction. This includes, for example, perceptiveness of others' internal states and moods; general ability to deal with others; knowledge about social rules and social life; insight and sensitivity in complex social situations; use of social techniques to manipulate others; perspective-taking; and social adaptation (Silvera et al., 2001). These components/abilities of social intelligence can be further categorized as being related to either cognitive social intelligence or behavioral social intelligence (Lievens and Chan, 2010), a topic we turn to next.

### Cognition and Behavior

Cognitive social intelligence involves social perception or the ability to understand or decode verbal and non-verbal behaviors of others. Behavioral social intelligence is the degree to which one can successfully interact or socialize with others (Lievens and Chan, 2010). For example, the components listed above could be sorted such that knowledge about social rules and social life, insight and sensitivity in complex social situations, perspective-taking, and perceptiveness of others' internal states and moods could all be considered manifestations of cognitive social intelligence. As complement to this, the components of social adaptation, use of social techniques to manipulate others, and the general ability to deal with others, could all fall under behavioral social intelligence (Silvera et al., 2001).

This distinction is important because socially intelligent behavior is contextual, and what would be considered most appropriate or the correct action to take may change depending upon various factors. Therefore, successful social interactions require not only the knowledge of what matters, but also the knowledge of “when” and “where” to engage certain social actions. A critical process in developing behavioral social intelligence is the ability to understand other agents' and their social interactions, then apply this knowledge in contextually varied interactions (Frankovský and Birknerová, 2014). As an example, culture would need to be considered as a factor across individuals because cultural variables, such as drives, distinctions, and behaviors, are culture-specific and affected by cultural-identity (Hofstede, 2019). Like all facets of social interaction, we learn about the contextual variability when embedded in, and receiving feedback from, the social environment. For example, we learn cultural aspects of social intelligence through our family and society; as we grow, these collectively teach us how to interact with others appropriately and we are reinforced on the behaviors that are socially intelligent through countless different social interactions. As we next describe, ToM allows an individual to reason about and interact with their environment, as well as cooperate more effectively with others (Cuzzolin et al., 2020).

### Theory of Mind in Humans

In the prior section, we addressed social intelligence at a higher level. Here we consider Theory of Mind (ToM), major component of social cognition referring to the set of processes that allow an agent to attribute mental states to others and successfully engage in social interactions with other agents (Cuzzolin et al., 2020). Research suggests that the absence of an ability to develop theory of mind can substantially limit social interactions. For example, it has been posited that individuals with autism experience difficulties with social interactions (and/or among other things) as a result of lacking ToM and the ability to attribute mental states to others (Bera et al., 2019). Theory of mind allows us to recognize other humans as possessing unique knowledge, beliefs, and desires, based on which they take intentional actions (Wellman, 2011). Rather than assuming other agents move in unpredictable, meaningless, or undirected ways, we use this ability to form a ToM of other agents to be able to reason about, interpret, explain, and predict their behavior (Perner and Lang, 1999; Lecce et al., 2015). ToM is a critical component of cognitive development. As they grow, children experience countless interactions, the outcomes of which become the basis for learning how to distinguish meaningful behaviors, and understand which behaviors are rewarded and which are punished (Hofstede, 2019).

Because it is so critical to cognitive development, ToM abilities show up in infants by the end of their first year. These are demonstrated when infants begin to exhibit gaze-following behaviors and start to acknowledge themselves and others as agents who commit intentional acts and are capable of subjectively experiencing the world (Wellman, 2011). Researchers have demonstrated that 18-month-olds can interpret and subsequently perform the actions that a researcher had “intended” to demonstrate but failed to. These can be fairly complex, for example, such as putting a block in a hole after watching an experimenter fail to put the block in its correct hole (Allen, 2015). This illustrates some of the roots of cognitive abilities, such as false-belief understanding, as the child is able to interpret the desired actions of the researcher rather than mimic or imitate their actions. To break this down conceptually, the toddler needs to, first, ascertain that the literal actions the researcher performed were not the intended/desired actions. Second, the toddler has to orient to the researcher's objective state of reality (i.e., how to properly use the toy). From this the toddler then executes the intended action. Toddlers are also able to demonstrate complex visual-perceptual activities indicative of a developing theory of mind. For example, at around 14 months, toddlers will follow another agent's gaze around a corner or barrier—even moving to gain visual information while checking back and forth to visually confirm the other agent is simultaneously experiencing a reality that is congruent with their own (Wellman, 2011). This demonstrates the emergence of the ability to discern whether an agent is aware of events and recognize that an agent may not be aware of key circumstances if their experiences of situations are not updated, and thus act in ignorance of the state of the world (Wellman, 2011).

Traditionally, this ability, called false-belief understanding, is considered a major developmental milestone not achieved until a child is about 4 years old (Helming et al., 2014; Scott and Baillargeon, 2017). False-belief understanding is typically assessed with elicited-prediction tasks, such as the well-known Sally-Anne task. In this task, children are told a story about two friends, Sally and Anne. They are asked to predict where Sally will look for a marble after Anne has moved it from the location it was placed by Sally. Because Sally did not see this change in location, she is unaware of the marble's location and, thus, has a “false-belief”. There are other tasks designed to assess more advanced theory of mind and other social cognitive processes. One example is a second-order theory of mind variation of the Sally-Anne task that asks the child about what a third-person saw and their beliefs (Perner and Lang, 1999). This more advanced task has the child attribute a theory of mind to a third person (“Grace,” a character added as an observer to the Sally-Anne scenario). Children who have successfully developed a second-order theory of mind false-belief understanding would be able to responds that Grace knows that Sally will think the marble is still in the basket. They can explain that Grace watched Sally leave, then saw Anne move the marble before Sally returned, so Grace knows that Sally did not see the marble moved and thus possesses a false belief of the location of the marble (Perner and Lang, 1999).

Adult measures of Theory of Mind reflecting more advanced development social-cognitive abilities and processes have also been developed and validated. One widely known example is the Strange Stories task, which requires the individual to infer the speaker's meaning in the story. This is done, not from the utterance, but from the context provided, such as facial expressions, preceding context, and social relationships (Jolliffe and Baron-Cohen, 1999). The strange stories task has been validated in teen and adult populations and has shown to differentiate levels of cognitive functioning and ToM ability in individuals (Jolliffe and Baron-Cohen, 1999). Additionally, there are a number of variations of the strange stories task that have been developed—such as strange stories in film (Murray et al., 2017). Other evaluations of ToM have also been adapted to have several variations, such as the Reading the Mind in the Eyes Task (RMET; Baron-Cohen et al., 2001).

### Summary

An individual's level of social intelligence, in addition to cognitive and emotional intelligence, is a strong predictor of performance in social interactions (Kaplan and Haenlein, 2019). Unfortunately, properly defining and capturing social skills has not proven to be a straightforward task for researchers. Although measures and tools, through which social intelligence can be quantified, have been developed, there is not an established, reliable tool to capture social intelligence. Prior researchers have attributed this lack of an established tool to deficiencies in existing methodologies of measuring social intelligence in humans (Silvera et al., 2001). For example, they often require interviewing multiple people (i.e., teachers, peers, supervisors, etc.) to assess a single individual, making each administration more difficult and time-consuming. Further, the standard methods of measurement (i.e., self-report, and behavioral assessment by observers) are often not correlated with each other (Silvera et al., 2001).

There are also fundamental problems in trying to compare methodologies in social intelligence to other kinds of intelligence testing. The use of self-rating methods to measure social intelligence at the level of the individual is markedly different from traditional evaluation techniques employed in intelligence testing (Kihlstrom and Cantor, 2000). Though social intelligence differs from traditional intelligence testing, this is likely necessary to comprehensively evaluate the various ways it is used in social interactions. Thus, methods for capturing social intelligence competency are often derived from multiple sources, including peer, superior, and self-ratings (Lievens and Chan, 2010). In sum, the many distinct components of social intelligence, and the variety of methods used to measure it, although complex, helps distinguish it from other forms of intelligence (Lievens and Chan, 2010).

## Socially Intelligent Agents

Understanding intentions is a complicated task with even humans varying in competence depending on their social intelligence. In human-machine interactions, failures can occur because AI has neglected the importance of social intelligence for gauging intentions. As detailed above, this requires an effective exchange of social information, which necessitates that the agent is able to understand the meaning of social information conveyed by humans and that it is able to convey its own interpretable social information—establishing bidirectional reciprocity in their understanding the meaning of social information (Wiltshire et al., 2014). This is even more complicated when considering contextual factors. That is, successful functioning in collaboration with humans will require ASI imbued agents learn social and moral norms and the implicit knowledge foundational to any social situation. Further, it will require that this prior knowledge can be drawn upon to infer what others feel and desire, and to predict their behaviors (Bennett and Maruyama, 2021).

For an agent to demonstrate social intelligence, it has to understand that intents, feelings, mental states, personalities, and other qualities of an individual can be embodied through behavior. Like humans, then, agents are provided with a continuous channel of cues and signals during interactions that are perceived and displayed. This can help cognitively ground interactions so that the intents of an individual's immediate actions may be inferred by others and that predictions may be made of their proximal and distal goals or future actions. More broadly, this information can be used to predict the nature and quality of social relationships and establish awareness of the overall atmosphere of an interaction (Wiltshire et al., 2014). Thus, engineering social cognition requires an understanding of how signals are embodied through cues in support of developing agents with social intelligence (Fiore et al., 2013; Wiltshire et al., 2014).

Therefore, ASI will need to be able to adhere to contextually-situated social structures (i.e., code of conduct, the format of communication, use of honorifics, etc.), to be able to recognize, interpret, and convey social signals, and, critically, to interpret how it may shape the social scenario through interventions. However, the required knowledge and capabilities of an ASI could be purposely limited in situated contexts by operationalizing social structures and norms, where the standards of socializing and impacts of social expression are mappable. Culture, for example, can be mapped to support generalizability in an ASI by modeling culture as a set of features with differing parameters related to quantified social norms. An example of a potential feature of culture is interpersonal distance ranges, based on observed proxemics of socially-situated relationships in a society (Hofstede, 2019). Others have worked on less tangible aspects of culture. For example, Khan et al. (2013) devised an algorithm approach for culture sanctioned social metrics (CSSMs) where action-impact functions are changed depending on context. In follow on work, Bölöni et al. (2018) developed a computational model of social norms. This integrated cultural values (e.g., politeness), and quantified them using the earlier developed CSSMs. Based on this, an agent is able to maximize the successful implementation and enaction of norms during social interactions by simultaneously considering how an action affects the self and peers. Modeling deep culturally informed sets of beliefs/knowledge is particularly relevant to scoping the situations in which an ASI is likely to facilitate teaming through active interaction or intervention. Considering the spectrum of tasks at which artificial agents excel, and where humans struggle, it is likely that ASI may provide maximum benefit to teaming scenarios where the need for socially informed interventions leverages the kind of computationally intensive problem-solving at which AI is already adept. Additionally, an ASI should be useful, viable, and applicable whether it is embodied or not. Embodiment of an ASI would require more design considerations, such as providing affordances for facial expression through digital or physical/mechanical display. It does not limit the social intelligence required for ASI. Rather it adds to possible capabilities for ASI, but the social components can exist even in a virtual, or drastically simplified physical environment (Hofstede, 2019).

Regardless of how an ASI is implemented, the agent will need to be capable of capturing, interpreting, and generating social data through the use of social cues and signals—development of which are a result of the efforts of the interdisciplinary field of Social Signal Processing (SSP). As discussed, next, this utilizes theory from social and cognitive sciences, blended with computer science and engineering, to establish mechanisms for recognizing and interpreting social signals and cues through socially-aware computing (Vinciarelli et al., 2009; Fiore et al., 2013; Wiltshire et al., 2014).

### Social Signals Processing

The intents, mental states, and other qualities of an individual are embodied through their behavior, which provides a continuous channel of signals that are perceived and generated between agents so that one may infer the intent of an agents present actions, as well as predict near and future goals (Wiltshire et al., 2014). The process by which humans interpret social signals from cues is typically an unconscious process that brings about, often spontaneous, understanding of social awareness in situations (Wiltshire et al., 2014). Research in Social Signal Processing (SSP), an interdisciplinary domain that seeks to create socially intelligent computers through the modeling, analysis, and synthesis of social cues and social signals (Vinciarelli et al., 2009), has focused on the kind of behaviors providing critical context to identifying the needs of another during joint action.

Humans, regardless of culture, use social cues such as language, voice, facial expressions, and body gestures to convey signals such as thoughts, emotions, and intentions to another agent who then senses, decodes, and interprets the communications (Joo et al., 2019). These signals may either be obvious through direct/unambiguous cues—such as if a human agent furrows their brow and states that they are confused about a particular aspect of a task. The signals may also not be obvious through indirect/ambiguous cues to an agent, who would not be able to decode and correctly interpret the meaning behind the social cues associated with the communication unless they possess the appropriate preceding knowledge. For example, a human might remark they are confused while vocal inflections and eye rolls indicate their statement is intended to be interpreted sarcastically; thus, the intended interpretation of the remark is contradictory to what is stated literally. These simple examples illustrate the varied ways cues and context interact to help an interactor make mental state attributions.

Social cues are the observable features of an agent utilized as potential channels of salient social information and are transmitted as a set of physical/physiological actions (Wiltshire et al., 2017). Social cues may be a behavior, trait, chemical trace, or action, but necessarily are a perceivable stimulus, which allows a person to interpret a signal containing within the social cues, or the contextualized meaning of a cue or combination of cues, which conveys social information in interaction (Poggi and Francesca, 2010; Wiltshire et al., 2014). Social signals are meaningful interpretations of cues, and contain emotional, cognitive, social, and/or cultural information (e.g., mental state attributions or attitudes) that allow for a largely unconscious production of social awareness (Wiltshire et al., 2014). Interpretation of signals often draws from prior knowledge and experiences of social cues that have been established as situated, context-dependent social norms (Hogg and Reid, 2006). Conceptually, social signals exist at a semantically higher level than cues and are sensitive to the combination of cues and how they are contextually-situated (Wiltshire et al., 2014).

Research in SSP has examined the nuances, variability, evolution, and combinations of these social signals (Pentland, 2007; Vinciarelli et al., 2008) in multiple ways. From the behavioral perspective, Fiore and colleagues used social cues and social signals as a foundation for social interactions between humans and robots. For example, they studied how social cues supporting ToM, when manifested in human-robot interactions, can change the mental state attributions made by humans. They showed that cues such as robotic proxemic behavior (e.g., how close it would come), altered perceptions of a social presence (see also Warta et al., 2018). Computationally, the processing of verbal social signals has seen advances in analytical techniques (i.e., natural language processing, automatic speech recognition), though non-word verbal cues (e.g., laugher, sounds like “um”, “eh”, “hmm”, etc.) have been difficult to automatically detect in long utterances. Some progress has been made where, for example, deep neural networks are able to classify non-word sounds using adaption evolution strategy (CMA-ES; Gosztolya, 2022).

Processing non-verbal interaction, in contrast, is still poorly understood, making it difficult to operationalize and understand the use of social signals and behavioral cues (Wiltshire et al., 2014; Joo et al., 2019). Non-verbal social cues are more difficult to process as there are few validated tools available, and much work is still needed to be able to consistently differentiate behavioral signals. There has been success in applying deep neural networks for the biomechanical analysis of human gait, demonstrating high enough accuracy in both classification and prediction of gait patterns in individuals to be suitable for clinical applications (Horst et al., 2019). Yet the study of body language, has shown little progress in this field. The vast majority of body language research still utilizes manual behavioral coding, but, with the advent of SSP, there has been research into automated coding of behaviors made possible through developments in computer vision (Joo et al., 2019). For example, Kachur et al. (2020) used a personality diagnostics neural network to predict personality traits, by taking the priors obtained from a computer vision neural network architectures (trained on facial features) as input, in a large dataset containing the questionnaires of 11,292 respondents and 28,230 associated photographs (Kachur et al., 2020). Best et al. (2016) used identification of social cues by humans, in a mental state attribution task, to train machine classifiers to make ToM like judgments. They were able to show how machine learning can develop models capable of classifying social interactions and demonstrate its potential to develop artificial social intelligence. More recent research has used computer vision and facial recognition technology to identify political orientation (Kosinski, 2021). Although, applied more generally, the capability for an algorithm to enable prediction of personal attributes from facial features has great implications for human-AI interactions by facilitating the perception of emotional states, personality, or other traits by machines, and use this information to adjust their behavior according to their understanding of what we are thinking, feeling, and doing (Kosinski, 2021).

Artificial social intelligence will be required to process the social data that is embedded in these social cues, both verbal and non-verbal (Ambady et al., 2000), and interpret the signals they receive to obtain the social information contained within to understand social and moral norms (Bennett and Maruyama, 2021). For example, phenomena such as attention, politeness, agreement, dislike, etc. are communicated through various social cues, that can include facial expressions, voice tone, gestures, and posture (Vinciarelli et al., 2008). Because social cues convey, either directly or indirectly, interpretable social signals that contain information about social actions, interactions, emotions, attitudes, and relationships (Poggi and Francesca, 2010), this understanding will foster effective interactions between agent and human. To help with this, a taxonomy of social cues and signals was defined by Wiltshire et al. (2014). This includes five categories of social cues which may be extracted and used to predict possible social signals: paralinguistic (voice prosody and non-language sounds), facial expression (motion and position of facial muscles), gaze (motion and position of the eyes and predicted sight-line), kinematics (motion, position, and posture of the body), and proxemics (use of interpersonal space) (Wiltshire et al., 2014, p. 90840F-4). Their development of a social cue taxonomy was motivated by the need to develop and integrate sensor capabilities into systems that can receive cues and interpret signals.

The ASI will need to be able to perceive and interpret behavioral, non-verbal social signal data as having a symbolic meaning that may be obvious or ambiguous, but still follows conventions established by social norms (Santoro et al., 2021), much in the same manner as verbal social signal data has been utilized. Further, using multiple sensor systems for social interactions is important as subtle meanings are conveyed through the combination of certain social cues (Joo et al., 2019). The essential differences between social cues are necessary to be able to sense, decode and interpret social signals and, depending on the context for which the agent will be applied (Hofstede, 2019).

Although there are machine learning solutions for identifying some of the spectra of signal patterns under a limited set of conditions, it is most likely that ASI will be least challenged when there is a limited, scoped set of signals they need to be able to interpret. A limited scope would afford the socially-immature ASI an initial foothold to build social intelligence in a particular area of application; but as ASI has more than just language and complex, deeply seated knowledge structures to contend with, ASI will likely need to be able to accurately interpret meaning from combinations of gestures and verbalizations. The development of ASI will require imbuing agents with social interaction abilities that, to be capable of successfully interacting, enable encoding, decoding, perception, and interpretation of a variety of social signals—a major goal, and challenge, for ASI research (Foster, 2019; Joo et al., 2019). However, a true socially intelligent agent should be able to engage with a human agent and derive their intentions, beliefs, goals (i.e., the ASI develops an artificial ToM), and use these models to anticipate what explanations or information may be relevant to the given human agent.

### Development of an Artificial Theory of Mind

Development of a theory of mind in humans when interacting with another, and how it is used to support that interaction, can be looked to as an analog in building an artificial theory of mind (AToM) for socially intelligent artificial agents (ASI). Young children learn from interactions with family and friends, and feedback “trains” their appropriate behavior in social situations. How an ASI is trained is an important issue as ASI will necessarily require the capabilities to develop an AToM, and use that to determine how to interact. Broadly stated, constructing an AToM can be done via development of models for agents, or by having agents learn via interactions. The former leaves agents with a capability of interacting in narrow contexts, while the latter requires significant amounts of interactions. In both cases, agents are limited in their ability to engage in socially complex situations (Hofstede, 2019). Thus, there is a growing need for ASI to be able to more broadly engage in emotional and social cognition, including utilization of a theory of mind, to ensure agents can act in novel, dynamic, or complex situations (Cuzzolin et al., 2020). We next briefly review some of the research addressing portions of this complex problem.

Humans use behavior modeling to intuitively understand what another is doing and engage in perspective taking to understand another's point of view (Chen et al., 2021a). That is, when humans engage in complex social interactions, they perceive others' latent characteristics and subliminal cues of mental states and use this to make inferences about knowledge or capability, facilitate interactions, and make predictions about future actions. To successfully demonstrate AToM, agents need to acquire at least rudimentary capabilities in these areas. Current agent-modeling approaches use reinforcement learning and imitation learning and simply focus on reproducing exhibited behavior, but do not account for internal mental states (Oguntola et al., 2021). Thus, they do not yet demonstrate capabilities to perceive social signals of human agents with whom they are interacting either through verbal cues (i.e., natural language processing) or non-verbal cues (behavioral modeling). Related to social signal processing, AI capabilities are still developing when it comes to processing and interpreting visual cues that link to symbolic social signals. This is the necessary precursor for making linkages between actors and objects, but difficulties remain in mapping natural language processing as symbols in an agents model to objects and situations in their environment (Kovalev et al., 2021) as might occur in situations requiring joint attention.

Interpersonal situations are complicated further when the agent's knowledge of the situation has to be created and maintained as well as updated. This requires an agent must create and maintain a model of the environment of interaction and the perceived social cues and their situated signals used in AToM attributions, etc. Further, this must be kept online with the ability to retrieve and reprocess it according to new contexts or queries so that the agent's knowledge of a situation may be used to generate multiple predictions and/or responses, and then be able to learn from feedback given some set of new outputs (Kovalev et al., 2021). Also necessary to development and updating AToM is the capability to integrate prior experiences for use in the current context. Here, the agent considers how current data was used for current model and would compare that to prior models based upon similar cues and signals. Essentially, the agent could infer consistency of behavior and use this integration of past and present observations to make predictions of future human behavior. Over time, the agent can become more accurate and better able to coordinate interactions. For example, a developed ASI would be able to able to determine when certain information is most beneficial to know and how to provide this information to a human collaborator, and either provide information on request or interpret the need for information from social signals in advance of direct communication. In the teamwork literature, the former is known as information pull and the latter is called information push. Studies show that high performing teams excel at information push (Orasanu, 1990; Stout et al., 1999). In the present context, this could involve perceiving the workload of a human collaborator, anticipating information needs, and providing necessary information in advance of requests without overwhelming the human agent.

Finally, what is needed are robust methods of assessment for determining the accuracy of AToM. The AToM of an ASI will need to be evaluated to assess the level of understanding it has of social signals; that is, has it learned the meanings behind symbolic social behaviors or simply mimicked behavioral sequences gleaned from observing prior interactions (Zadeh et al., 2019). How to assess this is an open research question. One option is to follow what is done with humans where, for example, AToM is indirectly inferred by inducing actions that reveal the ASI observer's understanding of the state of mind of an agent (Chen et al., 2021b). Another option is to develop agent assessments based upon ToM tests used to evaluate social intelligence in humans. For example, the advanced theory of mind task mentioned earlier (see section Cognition and Behavior), the Strange Stories task, has been used in research to evaluate individuals using two types of stories: theory of mind stories that require mental state attributions (such as persuasion, sarcasm, white lie, misunderstanding, or double bluff), and physical stories which require inferences on physical events (Lecce et al., 2015). Adapting this would require developing a large set of similar stories that could be used to training ML models able to distinguish differing mental and physical states. Then, agents could be tested whereby NLP is used to “read” stories from which attributions are made and appropriate actions decided. This aligns with other research that has shown question answering to be an effective way to train artificial agents on knowledge, though with agents still underperforming humans (Zadeh et al., 2019). One could argue that ASI should be subject to evaluation by tests of social intelligence intended for humans because they should be, at a minimum, capable of performing well on measures of general social intelligence (such as the Sally-Anne task or the strange stories task) if they are to interact with humans. Additionally, more complex NLP techniques could be developed to assess ASI in the context in which they will be employed. For example, this could be evaluated by considering agent understanding and use of the contextually-relevant meaning of mental-states verb activities. Called the metarepresentational verbs task (Lecce et al., 2015), this includes frequent use of mental-state terms and constructing the statement to embed a preposition in the main verb that may be true or false (e.g., Sally thought the marble was in the basket). Further, when considering a suite of the aforementioned measures, it might be possible for aggregated measures in task batteries can better assess components of ToM across varying levels of complexity (Hutchins et al., 2008). Unlike traditional evaluation of AI systems that are primarily based on the accuracy of its outputs, a psychometric-based evaluation of the level of social intelligence or ToM in agents will require going beyond numeric labels to properly assess whether the ASI possesses an AToM (Zadeh et al., 2019).

## Transparency and Trust in Human-Agent Interaction Through AToM

In this section, we connect our prior sections on social intelligence and theory of mind with a core attitude in teaming, that of trust. Humans can explain their intentions and decision-making processes in ways that are understandable and able to be affirmed by other human agents if those intentions are congruent with observable behavior (Bennett and Maruyama, 2021). In this way, the normally opaque mental state is transparent to the observer, and when actions proceed as expected, this helps foster trust. Over time, this helps us to understand the minds of other agents and affords us the ability to predict future behavior, verify its intent based on these models, and interpret what the behavior means. We can develop this trust by using multiple channels that make mental states transparent. We can listen to what a human agent says, and we can observe actions. In either case, we can predict that it will actively and intentionally seek behavior that aligns with its desires (Bennett and Maruyama, 2021). In this way, consistency in behaviors, helps develop accurate theory of mind (Shvo et al., 2020), and enables us to better interact, and establish efficient and effective communication (Rabinowitz et al., 2018), which are all crucial for collaboration with human teammates (Shergadwala and El-Nasr, 2021).

Considering the above in the context of human-agent teaming illustrates the challenge of trust and transparency as it relates to theory of mind. Said most succinctly, we can view this as the reciprocal formation of theory of mind whereby humans attempt to make attributions about the “mental state” of an agent teammate. We know that current AI systems are rather opaque, making it difficult for humans to comprehend reasoning. Further, this lack of transparency complicates the human's ability to determine intentions, or infer what guides agent decisions made and actions taken in the pursuit of goals. This lack of understanding of how AI systems come to their decisions, can lead to a lack of trust in the system. During high-risk situations (e.g., when errors can cause harm), this lack of trust is particularly problematic and may lead human teammates to reject the AI system (Akula et al., 2019; Rai, 2020; Papagni and Koeszegi, 2021).

The above challenges in trust and transparency illustrate well how the development of accurate AToM might alleviate these problems. First, ASI can support transparency by using AToM to predict how a human will respond to certain interactions. As described, these are based on internal models of contextually-relevant information and of prior interactions. ASI can use this to develop tailored interactions that make their intentions clear to users (Shvo et al., 2020). Agents with well-developed AToM are better able to communicate information that allows the user to understand why it took a certain action over another. From this, humans will be able to calibrate their trust of an agent and are more likely to develop an accurate ToM “of” the agent teammate. They will then be able to better predict when the system is expected to succeed, when the system is expected to fail, when to reasonably trust the system to perform as expected, and how to recover from an error (DARPA, 2016).

Our general point is that, to support trust in agents, facilitating the human's ToM of an artificial agent is equally as important as developing an agent's AToM of the human (Cuzzolin et al., 2020). The bi-directionality of these social cognitive processes will require both robot-to-human transparency and human-to-robot transparency (Lyons, 2013). As we've outlined, the opacity of agent decision-making processes renders humans unable to conceptualize how agents come to certain decisions. But the ability to understand and predict agent behavior is foundational to trust in ASI and critical to human-agent teaming. As described, there is a cascading set of consequences arising from diminished transparency. First, reduced trust in an agent will cause human users to not rely on the agent. This could occur even if the agent is capable. Second, this leads to under-use or misuse of the agent. Third, indiscriminate trust may lead to user complacency and failure to detect system failures due to over-reliance on the system (Alonso and De La Puente, 2018).

Trust and transparency is an important and developing area of research in human-agent teaming (e.g., Wright et al., 2019; Bhaskara et al., 2020; Barnes et al., 2021). Studies investigate a range of factors, from system design to team training, to understand how to improve trust in human-agent teaming. For example, Talone (2019) investigated the effect of transparency in reliability information on trust and appropriate use of an autonomous robotic teammate. He found that participants who were comprehensively informed of their robotic-teammates capabilities in different environmental conditions (e.g., materials that alter the robot's performance) during experimental training, were more likely to exhibit behaviors associated with appropriate reliance and increased trust. Those who were trained in reliability information of their autonomous robot's capabilities were more likely to indicate agreement with the robot's determinations of the presence of target objects in environments that did not contain materials known to impact the capabilities of the robot. Although not directly considering theory of mind “of” the agent, Talone's findings suggest that humans were better able to develop a model of their agent teammate and use it to better predict when performance would suffer depending on conditions in the environment. Thus, the training intervention made agent processes more transparent and helped the human teammate form a shared mental model (Mathieu et al., 2000) of agent capabilities. This suggests that training could be a method for developing a “theory of mind” of agent team members. That is, although not the objective of this study, methods like Talone (2019) are a potential path forward for turning the black-box of agent processes into a clear, transparent “glass-box” in which humans can interpret, comprehend, and anticipate agent behavior. Developing humans' ToM of an AI system within a human-agent team can potentially be accomplished by intentional information-sharing and contextually relevant training that aims to inform the human on factors that can affect an AI system's processing and decision-making. In short, increasing transparency can help establish theory of mind, which could then increase trust in the agent.

## Discussion

As AI progresses, interdisciplinary research is needed to sure machine agents are capable of collaboration. In this paper we have described how components of artificial social intelligence, in general, and artificial theory of mind, in specific, are foundational to human-agent teaming. In the context of human-human collaboration, lacking social intelligence hinders individuals in situations that involve the exchange of primarily social content without task-directed purpose or structure. AI, then, can only be expected to successfully engage in those sorts of scenarios effectively if they have social intelligence. By extension, a functional ASI could provide value in conditions where individuals with low social intelligence, or inadequate social intelligence (e.g., cross-cultural settings), may struggle. Guiding such research requires we more fully understand what are the key conditions (i.e., context, task, interpersonal) under which artificial theory of mind is most valuable in a team. Examination of social intelligence, its components, manifestation, and impact on human-human teaming, and in comparison, to human-agent teaming, helps lay the foundation for this research.

An additional need for such research is development of measures capable of assessing agent capability in mental state attributions, and how their implementation of these attributions, affects teams. We suggested various methods toward this end, including evaluation of agents using measures of social intelligence derived in the study of human social cognition. It should be noted that measurement of social intelligence in humans is still actively being researched as it is difficult to operationalize depending on context. Nonetheless, there are tasks that have been well-established and are consistent in evaluating social cognitive processes at various levels in individuals. Agents will need to be evaluated against similar criteria to their human counterparts—however, existing social intelligence and ToM assessments will need to be uniquely adapted for an ASI agent, such that they can receive, interpret, and generate social signal data. Further, the agent will also need to be able to develop and maintain dynamic mental models and AToM models of the other collaborating agents in the human-agent team as an online process that can be updated through processing social signal data (Briggs and Scheutz, 2012).

With the development of these more dynamic forms of ASI, they can eventually be implemented in settings potentially too complex for humans. Such situations might arise from mismatched mental models on a team (e.g., when members lack shared knowledge), or when *ad hoc* teams are formed (e.g., lack familiarity with each other). Here, ASI capable of diagnosing mental model mismatch, and intervening as needed, will help team more rapidly achieve coordination.

Future research implementing potential methodologies of modeling Theory of Mind in agents of humans, and framing explanations in ways that help to support human modeling of an ASI's Artificial Theory of Mind, will require an interdisciplinary effort between domains related to social sciences, computer science, and computational modeling.

## Author Contributions

JW conceived and prepared this manuscript. SF participated in the revision and guided the writing of this manuscript. FJ supervised and advised the writing of this manuscript. All authors provided critical feedback and helped shape this research. All authors contributed to the article and approved the submitted version.

## Funding

This research was sponsored by the Defense Advanced Research Projects Agency (DARPA) and the Army Research Office and was accomplished under Grant Number W911NF-20-1-0008. The views and conclusions contained in this document are those of the authors and should not be interpreted as representing the official policies, either expressed or implied, of DARPA, the Army Research Office, or the U.S. Government. The Government is authorized to reproduce and distribute reprints for Government purposes notwithstanding any copyright notation herein.

## Author Disclaimer

Any opinions, findings and conclusions or recommendations expressed in this material are those of the authors and do not necessarily reflect the views of DARPA, ARL, or the University of Central Florida.

## Conflict of Interest

The authors declare that the research was conducted in the absence of any commercial or financial relationships that could be construed as a potential conflict of interest.

## Publisher's Note

All claims expressed in this article are solely those of the authors and do not necessarily represent those of their affiliated organizations, or those of the publisher, the editors and the reviewers. Any product that may be evaluated in this article, or claim that may be made by its manufacturer, is not guaranteed or endorsed by the publisher.
